# Considering equity in global health collaborations: A qualitative study on experiences of equity

**DOI:** 10.1371/journal.pone.0258286

**Published:** 2021-10-07

**Authors:** Marlyn C. Faure, Nchangwi S. Munung, Ntobeko A. B. Ntusi, Bridget Pratt, Jantina de Vries

**Affiliations:** 1 Department of Medicine, Faculty of Health Sciences, University of Cape Town, Cape Town, South Africa; 2 Division of Human Genetics, Faculty of Health Sciences, University of Cape Town, Cape Town, South Africa; 3 Centre for Health Equity, School of Population and Global Health, The University of Melbourne, Melbourne, Australia; University of Cape Coast, GHANA

## Abstract

International collaborations have become the standard model for global health research and often include researchers and institutions from high income countries (HICs) and low- and middle-income countries (LMICs). While such collaborations are important for generating new knowledge that will help address global health inequities, there is evidence to suggest that current forms of collaboration may reproduce unequal power relations. Therefore, we conducted a qualitative study with scientists, researchers and those involved in research management, working in international health collaborations. Interviews were conducted between October 2019 and March 2020. We conducted 13 interviews with 15 participants. From our findings, we derive three major themes. First, our results reflect characteristics of equitable, collaborative research relationships. Here we find both relational features, specifically trust and belonging, and structural features, including clear contractual agreements, capacity building, inclusive divisions of labour, and the involvement of local communities. Second, we discuss obstacles to develop equitable collaborations. These include exclusionary labour practices, donor-driven research agendas, overall research culture, lack of accountability and finally, the inadequate financing of indirect costs for LMIC institutions. Third, we discuss the responsibilities for promoting science equity of funders, LMIC researchers, LMIC institutions, and LMIC governments. While other empirical studies have suggested similar features of equity, our findings extend these features to include local communities as collaborators in research projects and not only as beneficiaries. We also suggest the importance of funders paying for indirect costs, without which the capacity of LMIC institutions will continually erode. And finally, our study shows the responsibilities of LMIC actors in developing equitable collaborations, which have largely been absent from the literature.

## Introduction

International collaborations have increasingly become the research model of choice in global health research [1]. These collaborations usually include partners and institutions from high-income countries (HICs) and low- and middle-income countries (LMICs) [2]. Such international collaborations have the potential to generate new knowledge that can help reduce local and global health inequalities [3–8], offer opportunities for LMIC researchers to be involved in innovative research projects, and increase LMIC research capacities [6, 9, 10]. For HIC-based researchers, global health research collaborations may provide an opportunity to engage in socially responsible research [11].

While these are significant benefits of collaborations between HIC and LMIC researchers and institutions, there are concerns about the extent to which collaborations promote equality and fairness. In some instances, these collaborations have been labelled as neo-colonial [12] and potentially undermining equity [13–15]. And while HIC researchers may be well intentioned, they also benefit from attracting research funding to conduct research in LMICs [16, 17], access to and publishing on data which they would otherwise struggle to obtain, which subsequently allows for their career progression through recognition and promotion [16, 18].

Literature aiming to understand and foster equity in global health research has three main areas of focus. The first concerns normative accounts of why equity is essential [19, 20]. This work draws on political philosophy relating to social justice, which highlights the following practices as imperative for moving towards more just relations: mitigate unequal power relations that reproduce material and symbolic power differentials [21–23]; ensuring group recognition by considering the contributions and perspectives of all groups [22, 24–26]; promoting inclusive decision-making [22, 27–29]; promoting the health and well-being of those considered marginalized or disadvantaged [21, 30]; and ensuring self-development, which means making available opportunities for individuals to improve their capacities [31].

The second area of focus in literature discussing equity in global health research relates to the development of frameworks and metrics that operationalize principles and norms with the aim of standardizing, implementing, and measuring specific aspects of equity. One of the earliest guidelines developed was by the Commission for Research Partnership with Developing Countries (KFPE) [32]. More recent frameworks, such as the Partnership Assessment Toolkit developed by the Canadian Coalition for Global Health Research [33] recognised the lack of voices from the global South and have consequently adopted more inclusive approaches. Similarly, the Research Fairness Index, developed by the Council on Health Research for Development (COHRED), does not only provide practical guidelines but also offers a reporting mechanism for research organisations to assess and improve equity within partnerships [34, 35]. Generally, these guidelines and frameworks focus on various features of global health research, including capacity building, responsibilities within research projects, transparency and accountability to partners and funders, data sharing practices, dissemination of results and principles related to ownership of data and national development of research capacities [15]. While important, such frameworks may not provide adequate guidance on dealing with the practical challenges of achieving equity in collaborations [36].

The third area of focus concerns empirical studies exploring researchers’ experiences of working in international research collaborations. The studies in this focus area highlight the need for fair funding arrangements, including fairness in control over funding and decisions about how and where money is spent [1, 37–39]. This literature suggests that capacity building is critical to transfer skills [37, 40, 41] and grow the research independence of stakeholders in LMICs [39, 42]. Moreover, empirical studies underscore the need for fair research agreements, including shared decision-making between collaborators and LMIC actors’ inclusion as intellectual contributors to the collaboration [39, 41, 43]. Related to more equal divisions of labour, studies describe the importance of recognizing the contributions of LMIC actors [1], particularly through authorship. The invisibilization of LMIC authors in academic publications is especially glaring given bibliometric studies showing LMIC authors are either not listed as authors in some instances or are frequently not listed as first or senior authors despite research being conducted in LMICs and LMIC investigators leading key components of the research [44–46]. Trust and communication between research partners are also identified as necessary in this literature [1, 37]. Finally, these studies note that the health needs of LMIC communities need to be prioritized [38, 39] in global health research collaborations and that LMIC communities must benefit from research [1, 38, 47].

While normative accounts and practical guidelines make an important contribution to understanding what equitable collaborations should look like, empirical studies have often highlighted the practical challenges inherent to achieving them. Empirical studies can provide important insights from the experiences of those involved in developing equitable research collaborations. They could also inform policies, frameworks, and guidelines related to equitable research collaborations in global health. Yet, there are surprisingly few empirical studies on science equity, despite the significant increase in global health collaborations [48].

This study was conducted within the context of a larger study focussing on equity within the Human Cell Atlas (HCA) consortium. The HCA is an international scientific collaboration that seeks to create comprehensive reference maps of the cell states of all the cell types in healthy human bodies, characterized both in terms of stable properties (such as DNA sequence) and transient features (such as RNA expression and protein profiles). Although currently largely positioned in laboratories in North America and Europe, the ambition is for the Atlas to represent global diversity–in terms of where samples are drawn from and how scientists across the world are involved [49, 50]. Given the global scope of the study, we conducted a qualitative study with researchers and those involved in research management about their experiences of equity within the context of international health collaborations. Participants were either members of the HCA or had extensive experience working in international collaborations. Our study explored experiences of equity more generally and specific aspects related to the HCA. During our analysis, however, findings were more general and not exclusive to the HCA.

## Methods

### Study design, participants, and sampling

To gather evidence about equity in health research, based on the experiences of those involved in global health research collaborations, we conducted 13 interviews with 15 participants (scientists, researchers and research managers) between October 2019 and March 2020. In two instances, participants asked if they could be interviewed together with another colleague involved in global health collaborations. Initially, we recruited participants involved in the HCA consortium, primarily members of the HCA’s equity working group or who were present at HCA meetings that specifically discussed equity. To complement these interviews, we also recruited participants who were not part of the HCA but had extensive experience working in international collaborations. We thought the inclusion of those outside of the HCA would enrich our understanding of equity for the HCA and more generally. We also involved scientists and researchers and those occupying roles relating to research management, given that we were also interested in features and experiences of equity that were not exclusively related to research but also were critical to establishing equitable collaborations.

We relied on purposive sampling [51] because we were specifically interested in recruiting participants involved in the HCA or who had experience in international collaborations. We also sought to ensure a geographically diverse sample. Participants were based in Nigeria, South Africa, India, Bangladesh, Singapore, the United Kingdom, the United States and Switzerland. We had hoped to recruit more HCA members and had contacted several potential interviewees, however, it was challenging to find a suitable time to conduct interviews given participants’ busy schedules. Furthermore, the HCA is a grassroots organisation of basic scientists, with little in the way of identifying projects that belong to it, which made it difficult to identify HCA-affiliated researchers. That HCA researchers tend to be basic scientists–with possibly little experience in or need to reflect on the ethical aspects of their work–may also have compromised our ability to recruit HCA researchers for this project. After trying to recruit more HCA members, we decided to increase the number of participants not affiliated with the HCA. For participants who were not affiliated with the HCA, we also relied on purposive sampling to identify participants who had experience in working in international research collaborations. Table 1 summarises participant details.

**Table 1:** Overview of the geographic location, occupation, and HCA involvement of research participants in this study.

Details of interviews	Details of participants/interviews	
Number of interviews	13	
Number of participants	15	
Geographic location	LMIC	8
	HIC	7
Occupation of participants	Researchers	11
	Research management	4
HCA Involvement	HCA member	6
	Non-HCA member	9

### Research instruments

MCF and JDV developed the semi-structured interview guide with input from NSM and BP. Questions to participants covered a range of topics relating to their experiences of equity in international research collaborations, familiarity with or experience of the HCA, and bottlenecks or obstacles that stand in the way of achieving equity. This included describing what they understood equity to mean, their experiences of being involved in international collaborations, what they thought is necessary for collaborations to be equitable, and how issues of equity manifest in various aspects of the research process. While most of our questions related to their experiences and ideas about equity in international research collaborations, we had one section specifically asking all participants questions about equity and the HCA. While our interviews touched on the HCA, we found that interviewees’ responses (including members of the HCA) were not particular to the HCA. Therefore, we opted to present our findings in more general terms. When we asked participants about the HCA specifically, their responses were often generalizable and consistent with their responses to more generic questions about equity.

### Data collection

All interviews were conducted in English by MCF. Three of the interviews, with four participants, were conducted face to face and were audio-recorded. The remaining 10 interviews, with 11 participants, were conducted via an online platform and were audio-recorded. All interviews were transcribed verbatim. Interviews lasted between 45 and 90 minutes. While we had initially planned on including more participants, the emerging COVID pandemic and participants’ busy time schedules made it difficult to continue recruiting participants for this study. When we analysed the data we had, we realised that we had reached saturation on the main themes, meaning that participants repeated similar ideas related to previous interviews [52].

### Data analysis

Data analysis was iterative and commenced after the first interview; early themes were probed in subsequent interviews. A thematic approach was used to analyse the data, and themes were derived inductively [53]. Given that we were interested in participants’ experiences of equity and how they conceptualised equity, we relied on an inductive approach to our analysis. First, MCF read through the transcripts several times for data familiarisation and then used three transcripts to develop an initial list of open codes. Open codes were analysed to develop a hierarchical coding system together with NSM and JDV. MCF developed a codebook with definitions of each code and overall themes, and these were discussed with NSM and JDV. Once themes and codes were finalized, MCF, NSM, and JDV together coded the entire dataset. MCF collated and read through the coded dataset to verify the accuracy of the coding and consulted with JDV and NSM where any discrepancies arose. We used NVivo 12 to support data analysis [54]. In the section below, we have used anonymous interview codes. In each of the codes, the first letter indicates the occupation of the participants (G = researcher manager; S = scientists/researcher) and the last letter represents where the participant is based (N = global North; S = global South). The numbers in between the letters were randomly selected to distinguish interviewees.

### Ethics approval

This study was approved by the University of Cape Town, Faculty of Health Sciences Human Research Ethics Committee (641/2019). All participants were sent informed consent forms and interview guides and provided written consent before the interview.

## Results

Three major themes were derived from our analysis: 1) features of equitable collaborations; 2) why inequality persists; 3) who is responsible for equity.

### Features of equitable collaborations

#### Relational characteristics

The first theme in our results relates to features of equitable collaborations. Here participants reflected on both relational and structural aspects of equitable partnerships.

When describing relational characteristics, several interviewees described that equitable collaborations create a sense of co-ownership over the project. One participant noted:

…[W]here, the parties involved look at themselves as partners… the parties involved are co-creating knowledge. When the parties trust one another. When the parties are in what is called not a collaboration but a partnership. (S850S)

Notably, participants described that an equitable collaboration is not necessarily one that is equal. Instead, it is about “agreeing to something which we should all feel that it is fair” (S678N).

In addition to partnerships, participants also commonly used the terms ‘friendship’ and ‘creating a sense of belonging’, with one participant reflecting on this idea:

And this sense of belonging is what is the hallmark of equity. So, if you think of a project where there are multiple stakeholders and if every stakeholder feels a sense of belonging, that’s what I would term a project where equity is a hallmark… (S467S)

Prioritizing equity as an important value, even when it conflicts with other values such as expediency, was critical to building trust between individuals and institutions.

In addition, participants also described several structural features of equitable collaborations.

#### Structural characteristics

*Clear contractual agreements*. While relationship-building was a sign of equitable collaboration, participants also emphasized the importance of contractual agreements as necessary to building equity:

…if you want to create good partnerships, you need good contractual agreements, whether it is an informal agreement or MOU or a formal contract. But contracts are meant to regulate the quality and the equity or whatever you call the partnerships.” (G324N)

What transpires is that equity requires fairness, trust, and codified agreements to regulate collaborators’ expectations.

*Capacity building*. Our interviewees generally recognized capacity building as an important feature of equitable collaborations. While participants had varying perspectives on capacity building, they all described it as an ethical obligation and thus not optional. Capacity building was primarily framed as research training, such as training in specific scientific methods or statistical techniques or ensuring that students obtain graduate degrees. Importantly, our participants note that capacity building activities need to benefit LMIC collaborators. One participant was especially critical of simplistic forms of capacity building which may have the perverse effect of benefitting more powerful research partners:

…some capacity building programs run by Northern partners actually end up benefiting the Northern partners more than they benefit local people… Unless there is a deliberate effort to address this issue in research partnerships, it requires a lot of hard work and a lot of thought on how to do it. *(G238S)*

Capacity building can also focus on a transfer of skills or technological resources to individuals and groups. To promote equity, participants asserted that capacity building activities should aim to increase the research independence of LMIC partners. One participant noted:

I think capacity building is complex because it could involve just a technique, but I mean it should be more holistic in terms of developing the capacity to become independent researchers. To gain some intellectual and financial independence. *(G328S)*

While these forms of capacity building were seen as critical components of any equitable collaboration, participants also noted the necessity to expand the scope of capacity building beyond individual researchers or research groups to include institutional capacity building:

… part of it will be about developing other capacities that are needed to support research; it’s also about the project management, and the report writing, and the financial side of it and all that. (*G053S)*

From our data, both research-related and institutional forms of capacity are critical for equitable collaborations. Participants also observed that it would require a significant commitment from all parties, including funders.

*Inclusive division of labour*. Another important feature of equitable collaborations that we identified relates to a more inclusive division of labour. Many of our participants based in LMICs were particularly expressive about only working in collaborations where they were not merely seen as sample collectors but also expected to contribute intellectually to the research project:

Even if the funds might be coming from Northern partners, we want to be involved … as intellectual drivers of the projects as much as possible. And even to the same extent that our Northern colleagues are, and then the benefits accrued in terms of, let’s say, research outputs and publication. *(G328S)*

*Involving local communities*. Local communities’ involvement in the research process was also seen as a key feature of equitable collaborations. Many participants described that the inclusion of community members was essential to developing equitable relationships. One important reason given is that the entire research enterprise would not be possible without local participation. Our participants seemed to suggest that communities should be involved throughout the research process:

Like how we include them [communities] more, even designing the next questionnaire, instead of giving them the question, can we sit down with them, design it together? Can we spend more time with them, and can we include them, give them credit for the work? (S459S)

The suggestion seemed to be that communities should be empowered to participate meaningfully in decision-making around the research process:

…we do use community advisory boards, but there are a lot of issues in terms of how much of a voice community advisory boards really have… is it just to have a look at the consent form and say it’s okay but, how much of a voice and how much of a role do they play? And then even to identify who are your stakeholders within the community and how do you engage them really fruitfully. *(G053S)*

Participants also described that communities must benefit from research and applications developed from it. One participant noted that although basic research would generate few direct benefits for research participants, it is possible to design benefit-sharing approaches creatively and to include skills development and empowerment programmes or health education consultations and workshops. This could also be done, for instance, through preferential licensing arrangements, as one of our participants noted:

…We [research collaborators] agree, and it’s written that for those communities, these diagnostics that we are developing will be for them at production costs. There’s not going to be any profit from you producing it because their contribution resulted in the diagnostics, so they must get something back. And that’s how you can make equity. *(S850S)*

One participant noted that whilst developing benefit-sharing models with communities is important, it is complex and can have a perverse impact if not taken seriously:

I think it is important to avoid simplistic approaches to it because you can easily end up with a rather instrumental approach to it, with benefit-sharing although it starts from a good point of view, applied in the wrong way can begin to seem a bit like kind of ‘how do we buy our way into doing all we want to do’. *(S678N)*

### Why inequality persists

The second central theme from our data relates to why inequality persists in international collaborations. Our participants reported that one important reason was exclusionary labour practices that are a common feature of inequitable collaborations. In such practices, LMIC partners would typically be responsible for sample collection, while HIC partners would be responsible for intellectual aspects of the project, such as proposal or manuscript writing and data analysis. LMIC participants noted their exclusion could also be indirect, for example, by providing LMIC collaborators with insufficient time to achieve realistic milestones or develop appropriate budgets. A participant working in the research management office of a research-intensive African university reflected on how common this is:

Very often there are these very last-minute approaches… where a researcher comes to us and says, we urgently need this signed… our finance officers work with them to say this is not sufficient. Actually, you left out these costs, and you need to build these indirect costs for the institution, etcetera. And then the PI goes, but they are only offering us this much money, and we say well you need to go back and negotiate for more money, and this is a challenge … If they have literally been approached in the final week, there is very little they can do… *(G890S)*

Such exclusionary labour practices were partly attributed to structural challenges relating to some research funding schemes whereby institutions based in HICs are awarded large grants directly from funders and subsequently create sub-awards for LMIC institutions. One researcher reported on their own experience of this arrangement:

Like most of the organizations in the Global South are actually not directly funded. They become a sub-awardee, and the way that works is… We [researchers based in HICs] will tell you exactly what you do, we will tell you that these are the number of mothers we want to enrol, these are the number of children we want to enrol, this is the test we will do, and we don’t care about anything else, we just want data. That’s how most of the studies work. *(S459S)*

Such exclusionary practices risk perpetuating a relationship of dependency for LMIC researchers:

By not enabling them [referring to LMIC researchers] to do their data analysis, publishing the research in their name all of that, you are literally just getting them to collect the blood sample for you. Here… you are continuing to give them a situation where to survive they need your money, but they can never get better, that is another form of oppression in a way… (*G890S)*

Perhaps another reason for exclusion of LMIC researchers relates to how the research system values some practices and not others. For instance, the research system is set up to recognise and reward publications and grant funding, and often does not recognise or reward others, such as the extent to which a researcher promotes equity:

But the world we live in rewards one thing. And one thing only. Which is how many publications have you produced, and how much grant money have you brought in. *(S453S)*

Another important theme relating to inequitable collaborations is the prioritizing of donor interests. Participants reflected on how donors based elsewhere often influence or determine research agendas in local contexts. One participant asserted that these priorities were often also in the interest of profit:

Global health doesn’t change in two years, but they [funders] are always looking for silver bullets, probably to either fund a start-up or continue funding of a start-up they have already funded. Perhaps there is a vaccine that pharmaceutical companies are making, and they want to introduce the vaccine, and they want us to generate data for the vaccine…. So, I think there’s like they have individual agenda…. I think it’s all about capitalism. *(S459S)*

In this context, the respondent appears to be signalling that funders have considerable power in setting research agendas and situated such practices within larger global economic and cultural systems.

Another participant followed a similar logic in reflecting on how research projects may result in donor countries benefitting more than their counterparts in the global South, for example, through the concentration of resources and skills. Against this observation that some parties benefit from inequitable relations sits a concern that more powerful researchers and institutions are generally not held accountable for their actions. Various LMIC participants relayed experiences of feeling exploited yet having very little recourse to ensure their HIC collaborator was held to account, despite reaching out to the collaborator’s institution and the funder. One participant linked such impunity to how HIC researchers are often protected by their institutions:

I guess I’ve heard too many stories of institutions covering up really spectacularly bad behaviour. And I would not be optimistic that… institutions would act equitably… I think, unfortunately, the greater the status of an institution, the more it has to lose. *(S543S)*

Finally, some of our participants, especially those involved in research and grant management, also noted how not paying the total costs of research erodes institutional capacity to effectively manage research projects and provide a conducive environment for research to thrive:

The challenge is you end up in a vicious cycle. If you are chronically recovering insufficient indirect costs, you are never going to have properly maintained buildings, properly maintained finance support, enough legal staff to sign off contracts rapidly for you…And a lot of first-world institutions are getting in a lot more indirect costs to support their research and so they are able to offer more expensive facilities, bigger finance offices, bigger grants offices… *(G890S)*

Against that background, less powerful institutions in LMICs face an uphill battle, being allowed only to recover a fraction of the indirect costs associated with grants management while simultaneously needing to compete internationally with institutions receiving far more significant amounts and that are already better resourced.

### Who is responsible for equity?

Our third theme related to perspectives of who is responsible for ensuring equity. Here, somewhat surprisingly, our participants pointed to a range of LMIC stakeholders’ responsibilities alongside research funders.

#### Research funders

While our participants pointed out that funders are not a homogenous group and have different approaches, policies and constituencies to whom they are accountable, they argued that funders have an ethical responsibility to ensure equity is achieved in the projects they fund:

I think ethically they have got a responsibility. I think it is very easy for them to not pay attention to it. Probably because they are also overworked and tired, etcetera, but… if you are going to fund projects where big Northern partners are going to work with small Southern partners, I think as a funder, ethically, it is your responsibility to monitor how that is happening because otherwise, your money may not be going towards what your charitable endgame is. *(G89S)*

Specifically, our participants thought that funders needed to drive equity by ensuring sufficient resources are allocated to aspects of research and collaborations that could promote it, such as capacity building. Furthermore, our participants described that funders had a role to play in holding researchers who engaged in inequitable collaborations to account–but no concrete suggestions for how they could do so were made.

#### LMIC research institutions

LMIC research institutions were also seen as critical to ensuring equity. For example, one participant noted that African institutions are responsible for becoming “trusted institutions”, meaning that they can effectively conduct research and administer large grants in transparent ways. One participant asserted that whilst the challenges faced by researchers when conducting research in LMICs are located at the level of the researcher, these are often symptomatic of institutional challenges related to the under-development of capacities that are critical for research, such as financial, legal and administrative capacities. Other participants noted that institutions play a crucial role in protecting and supporting their researchers, for instance, when negotiating for more equitable terms in collaborations:

…when you fight as an individual, it is more difficult because people can easily overlook you as an individual…if they are serious and backup the local researcher, they can strengthen your hand. *(G238S)*

#### LMIC researchers

Our participants considered that researchers are the most obvious stakeholders responsible for ensuring that collaborations are equitable. Participants noted that while it is often true that HIC collaborators and funders set the research agenda, researchers based in LMICs have to assert their sense of agency:

…I think a degree of self-reflection is required when you are criticizing other groups or individuals… almost like being a victim…We are all subject to forces greater than ourselves, but you know we have some agency as well. We have to put a bit more energy into defining how we would like things to be, rather than just criticizing them. *(S678N)*

Similarly, one LMIC participant reflected that researchers based in LMICs have a choice about whether to get involved in collaborations that are unequal and, in some instances, exploitative, yet often do not realize that their contribution, by virtue of their access to communities and local knowledge, is indispensable to the success of the research project:

I also believe that we [LMIC researchers] should be the ones changing the narrative; we should be the ones setting the balance because we have the patients, we have the viruses and the parasite, and I don’t see why we shouldn’t just increase our bargaining power. Because without those viruses, without those patients, I don’t see how the research will go on. The problem is that our people [feel] so inferior, some people don’t believe in themselves… *(S850S)*

Lastly, LMIC research managers noted it was important for LMIC researchers to understand what is required for a collaboration to be equitable. For example, researchers need to be able to appropriately cost research in their context, consult with other actors to budget properly for indirect costs, and be part of the negotiations for equitable collaborations.

#### LMIC national governments

Participants also noted the role of LMIC national governments in funding research institutions and research. One participant, based in a HIC, emphasised the importance of LMIC governments funding sustainable research:

Local pride in your research capacity is a difficult thing to sustain in the political environments that exist in Africa. But you know, sooner or later, someone in government must feel they are proud of having a good, trustworthy international institution. *(G087N)*

Another participant also reflected on the importance of national LMIC governments investing in research that would result in scientific independence:

The respect comes with investing in the capacities that you have and trying to create that complementary expertise that creates equity. Otherwise, you are always under somebody’s thumb, and it just doesn’t work right. *(G987N)*

While it was not disputed that LMIC governments should fund research, others asserted that it was not entirely true that LMICs do not fund research and rely exclusively on the financial support of international actors:

I don’t agree with that hundred per cent because when they [HIC stakeholders] said you don’t put money into the research, I keep asking when you want to calculate that asset of those institutions in Africa, they are government-owned institutions for the most [part]. The government pays the salary for everybody working there. The government owns the infrastructure. Are those things quantified? I think we need to have that quantification; we need to have that discussion… *(S850S)*

One participant also noted that regional institutions, such as the African Union could play a key role in ensuring that LMIC researchers and institutions are protected and that research is regulated beyond the level of the nation-state. This was seen as an act of solidarity to ensure that all countries in a region leverage their collective power to foster equity.

## Discussion

International collaborations have become the standard model for global health research. Given the financial, geographical, technological, and status differentials between HIC and LMIC researchers and institutions, there is a concern that collaborations may entrench unequal power relations rather than interrupt or transform them [13, 15]. There have been attempts to provide corrective measures through normative accounts of what ought to be done and more practical guidance through the development of ethical frameworks. There are only a limited number of empirical studies published on the experiences of researchers and other stakeholders involved in international research collaborations. We conducted a qualitative study with scientists, researchers, and those involved with research management across LMIC and HIC contexts to understand their experiences of equity and what they thought contributed towards developing more equitable collaborations. From our results, we derived three significant themes related to equity within international health collaborations. The first refers to features of equitable collaborations, the second relates to reasons inequitable relationships persist, and the third focuses on the responsibilities of various actors for equitable relationships.

Our findings show that there are multiple components of equity. We highlight relational aspects of equity, such as a sense of belonging and trust. We also highlight structural elements which contribute to equitable collaborations. These include inclusive labour practices, contractual agreements, capacity building, research agenda setting, the inclusion of local communities from the onset of the research and ensuring that LMIC researchers are trained and effectively supported (by their institutions) in budgeting appropriately and paying for indirect costs. The dimensions of equity emerging from our findings, such as capacity building, fair funding arrangements, benefit-sharing, and trust, are supported by other empirical studies with similar findings [1, 37–39]. These findings are consistent with normative accounts of what constitutes equity in global health research collaborations and extend them.

Our findings suggest that community members’ inclusion as key stakeholders in the research process is essential for equitable collaboration. While community engagement is increasingly becoming a standard requirement for research funding, communities are generally perceived as beneficiaries of research and not always included at the outset of research projects, including as stakeholders who are part of setting research priorities and being involved in research design, for example. The necessity of including communities at all stages of the research process as a feature of equitable collaborations is akin to what Pratt and de Vries propose by drawing on shared health governance as a theory for delineating what more ethical and effective community engagement model should entail [55].

Additionally, echoing the findings by Crane [13, 56, 57], our findings suggest that funders paying indirect costs for LMIC institutions was seen as critical to developing equitable collaborations. Properly funded indirect costs are essential to the sustainable development of the administrative and financial capacity to manage large international grants. They also cover other essential aspects critical to fostering equitable collaborations, such as developing the legal capacities to negotiate fair contracts, paying for subscriptions to international journals, fostering innovation and other necessary infrastructural requirements. Our interviewees described that a chronic failure to recover the total costs of research undermines the institution and its ability to provide an environment where research can thrive. They outlined two challenges: inexperience in determining the full economic cost of a project and disagreements about who is responsible for funding institutional costs of research. Concerning the latter, while there has been some research on the responsibilities of funders, more research is required on balancing the responsibility of LMIC states and the long-term impact of colonialism and subsequent structural adjustment policies [58–60], with diverse types of international funders and their specific duties and obligations [61]. Furthermore, it would be important to understand better whether research that is led by LMIC researchers and funded by calls limited to them only [62] does lead to more equitable collaborations.

Moreover, what was surprising was that our participants rarely discussed the responsibilities of HIC researchers and institutions. While this could be because we did not include many interviewees based in HICs, another way to interpret this omission is that prioritizing equity would conflict with other values and practices which are incentivized in symbolic and material ways such as the status and reward associated with being listed as first or senior author and attracting grant funding which may be important for career advancement. To this point, our participants reflected on the larger structural challenges which shape how research is practised and its impact on different researchers depending on their geographic and institutional location. Moreover, we do not think this omission reflects that our participants thought HIC actors do not have an important role to play. Instead, it may reflect how intractable it may be to change a system that does not necessarily incentivize prioritizing equity and one in which benefits are derived from maintaining the status quo. Hedt-Gauthier et al. have noted how researchers based at HIC universities are promoted through exclusively focussing on factors such as publication numbers and citations without any attention given to roles played by their LMIC counterparts or the extent of the efforts undertaken to create more equitable collaborations [18]. HIC institutions can, however, begin to incentivize HIC researchers for developing equitable practices [18, 63].

Our participants did, however, discuss the role of LMIC research and institutions. While other studies have highlighted the responsibilities of HIC actors [64], there is a dearth of research on the role of LMIC actors. Our findings show that LMIC researchers need to become aware that they are legitimately contributing to the collaboration, and therefore also have the power to influence the nature of the collaboration. While the impact of unequal power relations was not denied, our participants noted that acquiescence on the part of LMIC researchers is also a function of choice and not necessarily compulsion. Related to this finding was the observation that LMIC researchers were also responsible for understanding how to cost and budget appropriately, with LMIC institutions providing the necessary training and support. Similarly, LMIC institutions were seen to be responsible for protecting their researchers, especially when negotiating for more equitable collaborations.

## Limitations

We had initially set out to interview members of the HCA Consortium but failed to identify an extensive range of stakeholders who were willing and able to speak on the topic of equity. We, therefore, transitioned the project to include senior researchers and others with substantive experience in international research collaboration, which shifted the focus of our project somewhat. While even the HCA-related interviewees spoke to us about more general issues relating to science equity–and not specific issues to the HCA–this seemed like a logical thing to do and did not seem to us to interrupt our study, but it does mean that our findings are not specific to the HCA but more generally to global health research. A second limitation is that we conducted interviews in English and thus excluded non-English speaking participants from other LMICs and HICs, who speak only French, Portuguese or Spanish. To develop a more comprehensive understanding of research equity in global health research collaborations, it is imperative that the views and experiences of non-English speaking LMICs are interrogated in future research.

## Supporting information

S1 File (PDF)

## References

[1] Parker M, Kingori P. Good and Bad Research Collaborations: Researchers’ Views on Science and Ethics in Global Health Research. PLoS ONE. 2016;11(10):e0163579. doi: 10.1371/journal.pone.0163579 27737006 PMC5063577

[2] Corbin JH, Mittelmark MB. Partnership lessons from the Global Programme for Health Promotion Effectiveness: a case study. Health Promotion International. 2008;23(4):365–71. doi: 10.1093/heapro/dan029 18835888

[3] Godoy-Ruiz P, Cole DC, Lenters L, McKenzie K. Developing collaborative approaches to international research: Perspectives of new global health researchers. Global Public Health. 2016;11(3):253–75. doi: 10.1080/17441692.2014.999814 25642809 PMC4819581

[4] Ward CL, Shaw D, Anane-Sarpong E, Sankoh O, Tanner M, Elger B. Defining health research for development: The perspective of stakeholders from an international health research partnership in Ghana and Tanzania. Developing World Bioethics. 2018;18(4):331–40. doi: 10.1111/dewb.12144 28470856

[5] Hanney SR, González Block MA. Building health research systems to achieve better health. Health Research Policy and Systems. 2006;4(1):10. doi: 10.1186/1478-4505-4-10 17087830 PMC1636053

[6] Airhihenbuwa CO, Shisana O, Zungu N, BeLue R, Makofani DM, Shefer T, et al. Research capacity building: A US-South African partnership. Global Health Promotion. 2011;18(2):27–35. Epub 2011/05/21. doi: 10.1177/1757975911404745 ; PubMed Central PMCID: PMC3322415. 21596937 PMC3322415

[7] Maziak W, Ward KD, Eissenberg T, Klesges RC, Keil U. The Syrian Center for Tobacco Studies: A model of international partnership for the creation of sustainable research capacity in developing countries. Promotion & education. 2004;11(2):93–7. doi: 10.1177/175797590401100206 15457871

[8] Van den Broucke S, Jooste H, Tlali M, Moodley V, Van Zyl G, Nyamwaya D, et al. Strengthening the capacity for health promotion in South Africa through international collaboration. Global Health Promotion 2010;17(2 Suppl):6–16. Epub 2010/07/17. doi: 10.1177/1757975910363923 . 20595334

[9] Horton D, Prain G, Thiele G. Perspectives on Partnership: A Literature Review2009. 111 p.

[10] Bradley M. North-South research partnerships: Challenges, responses and trends—A literature review and annotated bibliography. Working Paper 1, IDRC Canadian Partnerships Working Paper Series Ottawa: International Development Research Centre. 2007

[11] Yarmoshuk AN, Cole DC, Guantai AN, Mwangu M, Zarowsky C. The international partner universities of East African health professional programmes: Why do they do it and what do they value? Globalization and Health. 2019;15(1). doi: 10.1186/s12992-019-0477-7 31174554 PMC6555909

[12] Boshoff N. Neo-colonialism and research collaboration in Central Africa. Scientometrics. 2009;81(2):413. doi: 10.1007/s11192-008-2211-8

[13] Crane J. Scrambling for Africa? Universities and global health. The Lancet. 2011;377(9775):1388–90. doi: 10.1016/S0140-6736(10)61920-4 21074254

[14] Crane JT. Scrambling for Africa: AIDS, expertise, and the rise of american global health science. Ithaca: Cornell University Press; 2013.

[15] Costello A, Zumla A. Moving to research partnerships in developing countries. BMJ (Clinical research ed). 2000;321(7264):827–9. doi: 10.1136/bmj.321.7264.827 11009530 PMC1118627

[16] Chu KM, Jayaraman S, Kyamanywa P, Ntakiyiruta G. Building Research Capacity in Africa: Equity and Global Health Collaborations. PLOS Medicine. 2014;11(3):e1001612. doi: 10.1371/journal.pmed.1001612 24618823 PMC3949667

[17] Ishengoma JM. North-South Research Collaborations and Their Impact on Capacity Building: A Southern Perspective. In: Nossum THJ, editor. North-South Knowledge Networks: Towards Equitable Collaboration Between Academics. Cape Town and Bergen: African Minds and UIB Global; 2016. p. 149–86.

[18] Hedt-Gauthier B, Airhihenbuwa CO, Bawah AA, Burke KS, Cherian T, Connelly MT, et al. Academic promotion policies and equity in global health collaborations. The Lancet. 2018;392(10158):1607–9. doi: 10.1016/S0140-6736(18)32345-6 30496066

[19] Ruger JP. Global health justice. Public Health Ethics. 2009;2(3):261–75. doi: 10.1093/phe/php019.

[20] Pratt B, Hyder AA. Governance of transnational global health research consortia and health equity. The American Journal of Bioethics. 2016;16(10):29–45. doi: 10.1080/15265161.2016.1214304 27653398

[21] Powers M, Faden RR. Structural injustice: power, advantage, and human rights. New York: Oxford University Press; 2019.

[22] Young IM. Justice and the politics of difference. New Jersey Princeton University Press; 1990.

[23] Maldonado-Torres N. On the coloniality of being. Cultural Studies. 2007;21(2–3):240–70. doi: 10.1080/09502380601162548

[24] Fraser N. Social justice in the age of identity politics: redistribution, recognition, and participation. In: Peterson GB, editor. The Tanner Lectures on Human Values. Volume 19. Salt Lake City: University of Utah Press; 1998. p. 1–68.

[25] Fraser N. Rethinking recognition. New Left Review. 2000;3:107–20.

[26] Benhabib S. Toward a deliberative model of democratic legitimacy. In: Benhabib S, editor. Democracy and difference: contesting the boundaries of the political. New Jersey Princeton University Press; 1996.

[27] Gutmann A, Thompson D. Why deliberative democracy? New Jersey Princeton University Press; 2010 //.

[28] Daniels N. Just health: meeting health needs fairly. New York: Cambridge University Press; 2012.

[29] Ruger JP. Shared health governance. The American journal of bioethics: AJOB. 2011;11(7):32–45. doi: 10.1080/15265161.2011.568577 . 21745082 PMC3988676

[30] Ruger JP. Health and social justice. New York: Oxford University Press; 2010.

[31] Young IM. Inclusion and democracy. New York Oxford University Press; 2000.

[32] Guidelines for research in partnership with developing countries. Commission for Research Partnership with Developing Countries (KFPE), 1988.

[33] Afsana K, Habte D, Hatfield J, Murphy J, Neufeld V. Partnership assessment toolkit. Canadian Coalition for Global Health Research and International Development Research Centre, 2009.

[34] The COHRED Fairness Index for international collaborative partnerships. 2015. 10.1016/S0140-6736(15)60680-8 25890912

[35] Musolino N, Lazdins J, Toohey J, Ijsselmuiden C. COHRED Fairness Index for international collaborative partnerships. The Lancet. 2015;385(9975):1293–4. doi: 10.1016/S0140-6736(15)60680-8 25890912

[36] Matenga TFL, Zulu JM, Corbin JH, Mweemba O. Dismantling historical power inequality through authentic health research collaboration: Southern partners’ aspirations. Global Public Health. 2020:1–12. doi: 10.1080/17441692.2020.1775869 32496873

[37] Matenga TFL, Zulu JM, Corbin JH, Mweemba O. Contemporary issues in north-south health research partnerships: Perspectives of health research stakeholders in Zambia. Health Research Policy and Systems. 2019;17(1). doi: 10.1186/s12961-018-0409-7 30646902 PMC6334387

[38] Walsh A, Brugha R, Byrne E. "The way the country has been carved up by researchers": ethics and power in north-south public health research. International Journal for Equity in Health. 2016;15(1):1–11. doi: 10.1186/s12939-016-0488-4 27955670 PMC5153695

[39] Munung NS, Mayosi BM, De Vries J. Equity in international health research collaborations in Africa: Perceptions and expectations of African researchers. PLoS ONE. 2017;12(10). doi: 10.1371/journal.pone.0186237 29036174 PMC5643046

[40] Tindana P, Molyneux CS, Bull S, Parker M. Ethical issues in the export, storage and reuse of human biological samples in biomedical research: perspectives of key stakeholders in Ghana and Kenya. BMC Medical Ethics. 2014;15(1). doi: 10.1186/1472-6939-15-76 25326753 PMC4210627

[41] Moyi Okwaro F, Geissler PW. In/dependent collaborations: perceptions and experiences of African scientists in transnational HIV research. Medical anthropology quarterly. 2015;29(4):492–511. Epub 2015/05/14. doi: 10.1111/maq.12206 . 25800667 PMC4737198

[42] Muldoon KA, Birungi J, Berry NS, Ngolobe MH, Mwesigwa R, Shannon K, et al. Supporting southern-led research: implications for North-South research partnerships. Canadian Journal of Public Health. 2012;103(2):128–31. Epub 2012/04/26. doi: 10.1007/BF03404217 ; PubMed Central PMCID: PMC6973571. 22530536 PMC6973571

[43] Canario Guzmán JA, Espinal R, Báez J, Melgen RE, Rosario PAP, Mendoza ER. Ethical challenges for international collaborative research partnerships in the context of the Zika outbreak in the Dominican Republic: A qualitative case study. Health Research Policy and Systems. 2017;15(1). doi: 10.1186/s12961-017-0246-0 28946911 PMC5613490

[44] Hedt-Gauthier BL, Jeufack HM, Neufeld NH, Alem A, Sauer S, Odhiambo J, et al. Stuck in the middle: a systematic review of authorship in collaborative health research in Africa, 2014–2016. BMJ Global Health. 2019;4(5):e001853. doi: 10.1136/bmjgh-2019-001853 31750000 PMC6830050

[45] Rees CA, Lukolyo H, Keating EM, Dearden KA, Luboga SA, Schutze GE, et al. Authorship in paediatric research conducted in low- and middle-income countries: parity or parasitism? Tropical Medicine and International Health. 2017;22(11):1362–70. doi: 10.1111/tmi.12966 28857354

[46] Pingray V, Ortega V, Yaya S, Belizán JM. Authorship in studies conducted in low-and-middle income countries and published by Reproductive Health: advancing equitable global health research collaborations. Reproductive Health. 2020;17(1):18. doi: 10.1186/s12978-020-0858-7 32000792 PMC6993386

[47] Jentsch B, Pilley C. Research relationships between the South and the North: Cinderella and the ugly sisters? Social Science & Medicine. 2003;57(10):1957. doi: 10.1016/s0277-9536(03)00060-1 . 14499518

[48] Faure MC, Munung NS, Ntusi NAB, Pratt B, de Vries J. Mapping experiences and perspectives of equity in international health collaborations: a scoping review. International Journal for Equity in Health. 2021;20(1):28. doi: 10.1186/s12939-020-01350-w 33422065 PMC7796532

[49] Regev A, Teichmann S, Rozenblatt-Rosen O, Stubbington M, Ardlie K, Amit I, et al. The Human Cell Atlas White Paper. arXiv e-prints [Internet]. 2018 October 01, 2018. Available from: https://ui.adsabs.harvard.edu/abs/2018arXiv181005192R.

[50] Majumder PP, Mhlanga MM, Shalek AK. The Human Cell Atlas and equity: lessons learned. Nature Medicine. 2020;26(10):1509–11. doi: 10.1038/s41591-020-1100-4 33029017

[51] Patton MQ. Qualitative research & evaluation methods. 3rd ed. ed. Patton MQ, editor. Thousand Oaks: Sage Publications; 2002.

[52] Saunders B, Sim J, Kingstone T, Baker S, Waterfield J, Bartlam B, et al. Saturation in qualitative research: exploring its conceptualization and operationalization. Quality & Quantity. 2018;52(4):1893–907. doi: 10.1007/s11135-017-0574-8 29937585 PMC5993836

[53] Braun V, Clarke V. Using thematic analysis in psychology. Qualitative Research in Psychology. 2006;3(2):77–101. doi: 10.1191/1478088706qp063oa 32100154

[54] NVivo 11. 11 ed: QSR International Pty Ltd; 2015.

[55] Pratt B, de Vries J. Community engagement in global health research that advances health equity. Bioethics. 2018;32(7):454–63. doi: 10.1111/bioe.12465 30035349

[56] Crane JT, Andia Biraro I, Fouad TM, Boum Y, R. Bangsberg D. The ‘indirect costs’ of underfunding foreign partners in global health research: a case study. Global Public Health. 2018;13(10):1422–9. doi: 10.1080/17441692.2017.1372504 28920518

[57] Crane JT. Dreaming partnership, enabling inequality: administrative infrastructure in global health science. Africa. 2020;90(1):188–208. Epub 2020/03/11. doi: 10.1017/S0001972019001001

[58] Benton A, Dionne KY. International Political Economy and the 2014 West African Ebola Outbreak. African Studies Review. 2015;58(1):223–36. Epub 2015/03/16. doi: 10.1017/asr.2015.11

[59] Rowden R. The deadly ideas of neoliberalism: How the IMF has undermined Public health and the fight against AIDS. London: Zed Books; 2013.

[60] Pfeiffer J, Chapman R. Anthropological perspectives on structural adjustment and public health. Annual Review of Anthropology. 2010;39(1):149–65. doi: 10.1146/annurev.anthro.012809.105101

[61] Pierson L, Millum J. Health research priority setting: the duties of individual funders. The American Journal of Bioethics. 2018;18(11):6–17. doi: 10.1080/15265161.2018.1523490 30475176

[62] Pratt B, Hyder AA. Priority setting is more than resource allocation: reflecting on the content of funders’ duties and their implications for current practice. The American Journal of Bioethics. 2018;18(11):27–30. doi: 10.1080/15265161.2018.1523503 30475187

[63] Yap B II. Is Africa part of the partnership? Medicine Anthropology Theory. 2018;2(5). doi: 10.17157/mat.5.2.527

[64] Ruger JP. Ethics and governance of global health inequalities. Journal of Epidemiology and Community Health. 2006;60(11):998–1002. doi: 10.1136/jech.2005.041947 17053290 PMC2465483

