# Supplementary material for: Considering equity in global health collaborations: A qualitative study on experiences of equity
Source: PLoS One. 2021 Oct 7;16(10):e0258286. doi: 10.1371/journal.pone.0258286 (PMC8496851; doi:10.1371/journal.pone.0258286)
Supplement: S1 File — (PDF) [file pone.0258286.s001.pdf]

Interview Schedule: Expert Interviews

Study title: Developing a conceptual and empirical guide to Equity and the Human Cell Atlas Consortium

1. **Experience in international scientific collaboration**
   a. Briefly, could you describe your area of expertise (scientific) and your position
   b. Prompt: experience in international collaboration
      - ➢Number of collaborations, with which countries/institutions, involved in HCA?
   c. How did you get involved in this/these collaboration(s)?
      - ➢Importance of networking, self-initiated?
2. Have you been in a situation where there were disagreements about issues related to equity between collaborators? If so, how were these dealt with?
   a. Overall, what has been your experience of international collaborations?
      - ➢Equitable, exploitative, helicopter research?

Understanding of equity

3. What do you understand equity to mean in relation to scientific collaboration?
4. What informed your thinking about equity in this way?
5. Which stakeholders should be involved in discussions related to equity?
6. What is the relationship between equity and power? How/when is power shared equitably?

Equity and governance

7. How is equity relevant to the governance of research, and where does it manifest?
   a. Data collection
   b. Recruitment
   c. Who sets the research agenda versus who does the research
   d. Location of research
   e. Decision making, shared decision-making
   f. Control over resources & funding
   g. Research capacity development
   h. Knowledge translation (research uptake)
8. Who is accountable for ensuring equity in collaboration (i.e. funders, government, researchers)?

Equity and recruitment

Equity for communities and participants

9. I now want to ask you something about how we can promote equity locally. In your view, what needs to be done to ensure that scientific research is equitable for the participants? (or: how is equity relevant for the communities/participants that are enrolled in research?)
10. What role do benefit sharing arrangements play in promoting equity?
11. What could the HCA do to promote equity for research participants and communities?

Future

1. What would a fully equitable HCA/scientific collaboration in your field look like?
    a. Prompt: key features (shared decision making, shared resources), which domains (recruitment, governance)
2. Who should be accountable for ensuring equity in collaborations?
3. Are you aware of any mechanisms or tools that can be introduced to promote equity in collaboration?
